# Assessment of a molecular diagnostic platform for integrated isolation and quantification of mRNA in whole blood

**DOI:** 10.1007/s10096-015-2470-2

**Published:** 2015-08-23

**Authors:** C. H. van den Kieboom, G. Ferwerda, I. de Baere, H. Vermeiren, R. de Groot, R. Rossau, M. I. de Jonge

**Affiliations:** Department of Pediatrics, Laboratory of Pediatric Infectious Diseases, Radboud University Medical Center, Nijmegen, The Netherlands; Radboud Institute for Molecular Life Sciences, Nijmegen, The Netherlands; R&D Department, Biocartis N.V., Mechelen, Belgium

## Abstract

Implementation of point-of-care tests may facilitate the health management of infectious diseases by reducing the timeframe on pathogen identification and host response measurements, allowing for immediate diagnosis and guided clinical intervention. In this feasibility study, a novel totally integrated and fully automated real-time polymerase chain reaction (PCR) platform (Idylla™, Biocartis) was assessed to determine the mRNA expression levels of multiple genes from 1 mL of whole blood. To this purpose, a sample-in result-out assay, including mRNA extraction and RT-qPCR-based detection, was ported to the platform. The genes used (matrix metallopeptidase 9, olfactomedin 4, NB1 glycoprotein and lipocalin 2) were previously identified as predictive for severity of disease caused by infection with respiratory syncytial virus (RSV). The reproducibility and robustness of the prototype assay was determined using the blood samples of 21 healthy donors. The data showed that the Idylla™ platform allows for a fast and user-friendly determination of the relative expression levels of the four selected mRNA markers.

## Introduction

Infectious disease diagnostics changed profoundly over the past decades due to the rapid evolution in the application of molecular biological techniques, such as RT-qPCR. In this paper, a new approach to infectious disease diagnosis is explored based on the host gene expression of four selected mRNA markers predicting infectious disease severity [1–4]. The analysis of host gene profiles offers tremendous possibilities for the identification of markers of disease severity and, eventually, to predict the course of disease [5]. Parallel to the increased interest in host gene expression is the demand for rapid point-of-care testing.

We here assess the performance of a diagnostic platform (Idylla™, Biocartis) offering fully integrated nucleic acid isolation and multiplex RT-qPCR-based quantification. This sample-in result-out platform does not require specific expertise or molecular biological skills and allows for a minimum number of handlings before starting the assay run. With its short turn-around time and fully contained design, it is perfectly suited for rapid and safe decentralised diagnostics at emergency units, clinical departments or, ultimately, the general practitioner’s office.

The main aim of this study was to develop a prototype assay on the Idylla™ platform starting from 1 mL of EDTA whole blood measuring the relative expression levels of four mRNA markers specific for the severity of respiratory syncytial virus (RSV) infections in whole blood. These markers are: matrix metallopeptidase 9 (MMP9), olfactomedin 4 (OLFM4), NB1 glycoprotein (CD177) and lipocalin 2 (LCN2). They were all significantly upregulated during disease and were verified using peripheral blood mononuclear cell samples collected in a large clinical study [6].

## Materials and methods

### Sample collection

Whole blood samples were collected from healthy adults who were enrolled following informed consent. The cohort consisted of 21 subjects (52.4 % males), with an average age of 32.5 (20–44) years. Subjects who used any type of medication were excluded. The clinical information available to the study subjects included a description of the risks and benefits of participation and safe handling and protection of all data. We communicated to all subjects that there was no obligation to participate and that declining to participate or leaving the study had no adverse consequences, and written consent was obtained.

Whole blood samples were collected in EDTA vacutainer tubes (BD, USA). The samples were kept at room temperature and immediately transferred to the laboratory, where they were processed on the Idylla™ platform in the sequence of sampling.

### Idylla™ platform

**Fig. 1:**
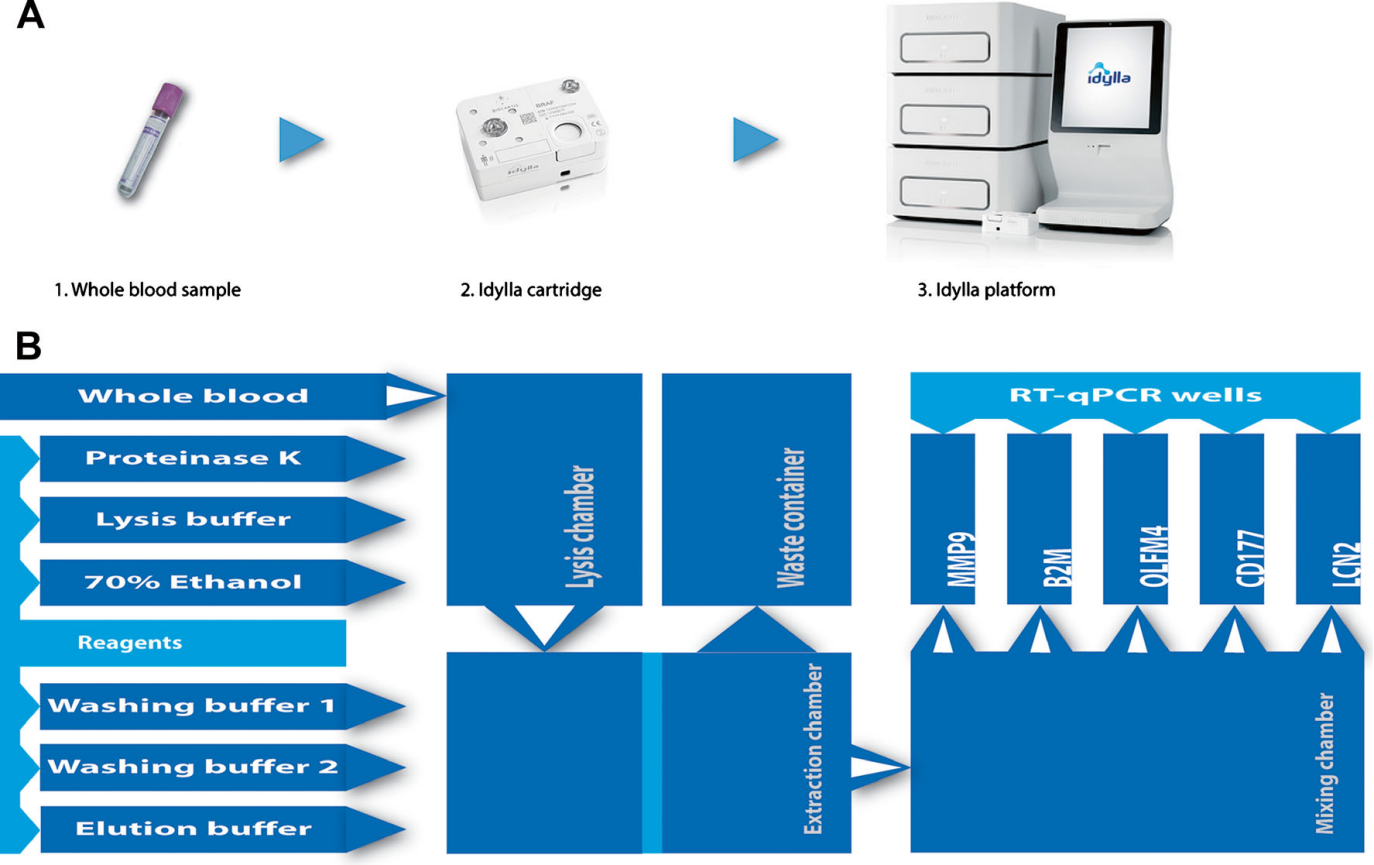
**a** The diagnostic processing sequence for use of the Idylla™ platform; from left to right: EDTA vacutainer tubes are used for blood collection (*1*), 1 mL of whole blood is introduced into the lysis chamber of the disposable cartridge (*2*), the cartridge is inserted into the instrument to run the automatic mRNA isolation and quantification procedure (*3*). **b** Schematic representation of the cartridge components, the prefilled reagents and the processing steps taking place in the cartridge, where the *white arrows* indicate the path of mRNA

Idylla™ is a fully automated sample-in result-out molecular high-precision diagnostic system consisting of one or more stackable instruments, a console and a disposable cartridge prefilled with all the necessary reagents for nucleic acid extraction and amplification and that is able to produce polymerase chain reaction (PCR)-based results from a single sample in about 2 h or less, depending on the complexity of the test, with a hands-on time of about 2 min (Fig. 1a). After addition of the clinical material in the cartridge and loading the cartridge into an instrument slot, all process steps are carried out automatically.

### mRNA isolation and quantification in Idylla™

One mL of EDTA whole blood was added into the lysis chamber of a research prototype cartridge prefilled with extraction reagents. The primers and probes from the respective TaqMan gene expression assays (Applied Biosystems, USA) were pre-spotted in each of the PCR wells (see Fig. 1b). After the addition of 100 μL RT-qPCR buffer to the mixing chamber, the cartridge was closed and added to a slot of the Idylla™ instrument and the automatic run was started, including the following steps. Firstly, 100 μL (20 mg.mL^−1^) proteinase K (Macherey-Nagel, Germany) was added to the blood sample and homogenized. Subsequently, 1 mL 3 M guanidinium-based lysis buffer (pH 7.3) was added to the mixture and, once again, homogenised by pumping the solution back and forth in the cartridge. Then, 1 mL 70 % ethanol was added to the lysate and, after mixing, the whole mixture was pumped over a silica membrane in the extraction chamber. Subsequently, the membrane was washed by pumping in 300 μL wash buffer 1 (RB2, Macherey-Nagel, Germany), followed by three times 300 μL wash buffer 2 (RB3, Macherey-Nagel, Germany) over the membrane. The residual ethanol was removed from the silica membrane by heating the membrane for 6 min. The nucleic acids were eluted in about 100 μL 10 mM Tris-HCl pH 7.3 and transported to the mixing chamber. After mixing the eluate with the RT-PCR buffer (95.25 μL iTaq universal probes one-step buffer and 4.75 μL iScript, both from Bio-Rad, USA), 25-μL aliquots of the mixture were pumped into the PCR wells each containing a primers and probe mixture of one of the biomarkers MMP9, OLFM4, CD177, LCN2 and B2M. The TaqMan gene expression assays (Applied Biosystems, USA) were validated on RNA specificity before a selection was made for use in the Idylla™ cartridge. The assays MMP9 (Hs00234579_m1), OLFM4 (Hs00197437_m1), CD177 (Hs00360669_m1), LCN2 (Hs01008571_m1) and B2M (Hs00187842_m1) were used at 120 % of the manufacturer’s advised concentration. The RT-PCR programme consisted of an initial incubation time of 10 min at 50 °C, followed by 2 min at 95 °C and 51 cycles of 10 s at 95 °C and 20 s at 60 °C. The Cq values were determined for each individual RT-qPCR reaction and, subsequently, the data were normalised by subtracting the Cq value of the housekeeping gene B2M, resulting in ΔCq values.

### Statistical analysis

To assess the normality of the results, the D’Agostino–Pearson omnibus normality test was used.

## Results

### TaqMan gene expression assay validation

The mRNA specificity of the different TaqMan gene expression assays were assessed to exclude background signals from residual DNA, since the protocol does not include a DNase treatment (data not shown). This evaluation led to the selection of specific primers and probe sets to measure the expression of the housekeeping gene B2M and the target genes CD177, LCN2, MMP9 and OLFM4 in our integrated assay on the Idylla™ platform.

### Biological variation

**Fig. 2:**
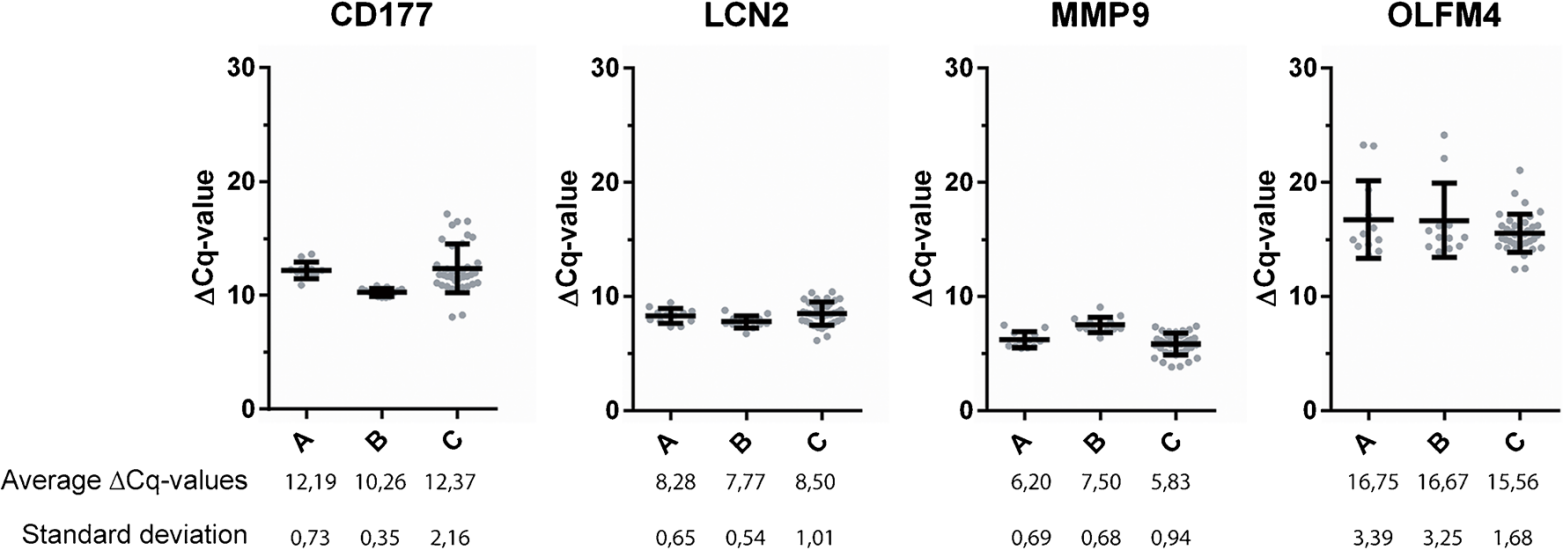
Normalised mRNA expression [mean ± standard deviation (SD)] of the measured targets for donor A (*A*) (*n* = 12), donor B (*B*) (*n* = 13) and 19 donors (*C*) [CD177 (*n* = 37), LCN2 (*n* = 38), MMP9 (*n* = 38) and OLFM4 (*n* = 36)]. Below the graphs, the average ∆Cq values and SDs are given

Whole blood of 19 healthy adults was analysed in duplicate and ∆Cq values and standard deviations (SDs) were calculated (Fig. 2). The ∆Cq values for all targets are normally distributed (*p* = >0.05), except for OLFM4 (*p* = <0.01).

### Technical variation

Multiple whole blood samples of two healthy adults, respectively donor A (*n* = 13) and donor B (*n* = 14), were analysed (see Fig. 2). For each of the donors, one run did not generate any values, so these runs were excluded from further analysis. In three runs of both donors, OLFM4 was not detected. For these, a Cq value of 51 was used for further analysis. The ∆Cq values were analysed on distribution, besides OLFM4 of donor B (*p* = <0.05), with all values having a normal distribution (*p* = >0.05).

## Discussion

In this feasibility study, we assessed the Idylla™ platform for host gene expression determination in whole blood. To this end, a fast all-in-one protocol was developed and optimised on this platform to isolate mRNA from 1 mL of whole blood, followed by a cartridge-compatible one-step RNA specific RT-PCR protocol for the four mRNA markers (CD177, LCN2, MMP9 and OLFM4) and the housekeeping gene (B2M).

This protocol was validated on whole blood samples from healthy individuals. Firstly, the biological variability for the four markers was determined using blood samples from 19 individuals. On average, MMP9 showed the highest expression levels, with a ∆Cq value of 5.83, followed by LCN2 and CD117, with average values of 8.50 and 12.37, respectively. With an average ∆Cq value of 15.56, OLFM4 showed the lowest expression, which implicates an almost 50,000-fold lower expression than B2M in blood of healthy individuals. This low expression is most likely the reason why OLFM4 was missed in about 5 % of the runs. The SDs for CD117 and OLFM4 are particularly high (2.16 and 1.68, respectively) as compared to LCN2 and MMP9 (1.01 and 0.4 respectively). For CD177, this most probably reflects the natural biological variation among individuals. In the case of OLFM4, this can also be attributed to the fact that its expression levels are close to the detection limit of the system.

Secondly, the technical variability was determined using multiple expression measurements of two donors (A and B). For both donors, the SDs obtained for CD177, LCN2 and MMP9 (between 0.35 and 0.69) were considerably lower than those obtained for the 19 samples of different individuals. This indicates that the technical variation is less than the biological variation among individuals. The opposite was observed for OLFM4, but this is due to the fact that, for each donor, this marker was missed three times, resulting in substantially increased SDs, because of these outliers.

In conclusion, the data demonstrate that the Idylla™ platform is a fast and highly convenient system able to measure simultaneously the relative expression levels of different mRNA markers directly in 1 mL of EDTA whole blood. In this study, only four markers have been used, but the multiplexing capability of Idylla™ has the potential to detect up to 30 markers simultaneously. The sensitivity of the assay needs to be further improved in order to also reliably measure low abundant mRNAs (such as OLFM4). Furthermore, to demonstrate the real clinical value of the assay, additional studies are needed using larger cohorts, including blood samples from diseased individuals.
